# Associative Learning during Early Adulthood Enhances Later Memory Retention in Honeybees

**DOI:** 10.1371/journal.pone.0008046

**Published:** 2009-12-02

**Authors:** Andrés Arenas, Vanesa M. Fernández, Walter M. Farina

**Affiliations:** Grupo de Estudio de Insectos Sociales, IFIBYNE-CONICET, Departamento de Biodiversidad y Biología Experimental, Facultad de Ciencias Exactas y Naturales, Universidad de Buenos Aires, Pabellón II, Ciudad Universitaria, Buenos Aires, Argentina; Freie Universitaet Berlin, Germany

## Abstract

**Background:**

Cognitive experiences during the early stages of life play an important role in shaping the future behavior in mammals but also in insects, in which precocious learning can directly modify behaviors later in life depending on both the timing and the rearing environment. However, whether olfactory associative learning acquired early in the adult stage of insects affect memorizing of new learning events has not been studied yet.

**Methodology:**

Groups of adult honeybee workers that experienced an odor paired with a sucrose solution 5 to 8 days or 9 to 12 days after emergence were previously exposed to (i) a rewarded experience through the offering of scented food, or (ii) a non-rewarded experience with a pure volatile compound in the rearing environment.

**Principal Findings:**

Early rewarded experiences (either at 1–4 or 5–8 days of adult age) enhanced retention performance in 9–12-day-conditioned bees when they were tested at 17 days of age. The highest retention levels at this age, which could not be improved with prior rewarded experiences, were found for memories established at 5–8 days of adult age. Associative memories acquired at 9–12 days of age showed a weak effect on retention for some pure pre-exposed volatile compounds; whereas the sole exposure of an odor at any younger age did not promote long-term effects on learning performance.

**Conclusions:**

The associative learning events that occurred a few days after adult emergence improved memorizing in middle-aged bees. In addition, both the timing and the nature of early sensory inputs interact to enhance retention of new learning events acquired later in life, an important matter in the social life of honeybees.

## Introduction

Behavioral responses of animals are continuously adjusted to their changing sensory environment. This phenomenon, however, is much more pronounced at early stages of life when experiences cause long-lasting and sometimes irreversible effects on behaviors [1]. In rodents, it has been shown that early experiences such as social deprivation or impoverished rearing environments have life-long negative consequences on cognition [2], [3]. In humans, it is known that speech development depends primarily on the presence of appropriate stimuli in the environment. The absence of speech models in children might negatively affect the acquisition of the language [4], whereas the exposure to a multilingual environment seems to improve learning performance [5].

In Arthropods, changes in the environment during the early stages of the adult lifespan can also affect behaviors at later stages [6]. For instance, in the ontogeny of nest-mate recognition in highly social insects, it has been shown that young worker ants are able to learn the odor of their social environment just after emergence, which strongly influences the recognition of colony members [7]–[9]. In the same way, in honeybees, olfactory cues paired with a reward shortly after emergence can be remembered when bees reach ages at which they initiate tasks outside the nest [10]. On the contrary, chemosensory experiences such as odors exposed as volatiles in the rearing environment, can reduce the conditioned response when this odorant cue is paired with a reward in a later conditioning [11], [12]. However, whether early experiences such as exposure to or learning of odorants during precocious periods improve retention of new learning events in insects is unknown.

Recent studies have shown that the behavior of an adult insect is more plastic at early ages than previously thought [10], [13] and thus that the time during which early experiences can shape later behavior is greater a few days after emergence than at any other adult ages [10]. When honeybees are reared under social deprivation within the laboratory, precocious learning of a particular odor associated with reward during a relatively short time window (when bees are 5–8 days of adult age) results in a better olfactory retention at an older stage (when bees are 17 days old) than when the same experience occurs immediately after this period (when bees are 9–12 days old). Interestingly, this difference is not observed when bees are reared within the colony [10], thus suggesting that a wide range of multi-modal stimulations may contribute to alter the levels of response between these age classes. Due to this non-linear effect of early olfactory experience, honeybees represent a good insect species to study whether precocious odor experiences improve the retention of a conditioned response (CR) that was acquired later in the adult life. Then, olfactory memories established after the first week of the adult worker (e.g. 9–12 days of age) could be better retrieved after an appropriate input of early stimulation.

Besides, the fact that retention of olfactory memories is age-dependent and not time-dependent [10] becomes particular interesting in honeybees, since they exhibit an extraordinarily tuned division of labor based on age polyethism [7]. In these insects, individuals of the worker caste progress from inside- to outside-nest tasks, a process that is accompanied by anatomical and functional changes [14]. Newly emerged bees start cleaning the cells or taking care of the brood, while middle-aged bees handle and store food until they initiate foraging from the third week of the adult stage [15]–[17]. To this end, the changing contexts at which the workers are exposed during adulthood make bees an excellent case to study the role of precocious sensory stimuli on later behavior.

To test this issue, we evaluated memories established in 9-12-day-old bees that were previously exposed to i) an odor-rewarded experience or ii) a non-rewarded experience either immediately after emergence (1–4 days of age) or a few days after emergence (5–8 days of age). Long-term memories were quantified during five consecutive testing events by presenting the conditioned stimulus (CS) alone (i.e. without reinforcement), a procedure that was expected to cause extinction [18].

## Materials and Methods

### Study Site and Animals

The experiments were carried out in the 2007/2008 summer season in the experimental field of the School of Exact and Natural Sciences of the University of Buenos Aires, Argentina (34° 32'S, 58° 26'W). Worker bees with a known age were obtained from sealed brood frames placed in an incubator at 36°C, 55% relative humidity and darkness. Emerged workers were collected in groups of about 100 bees and confined in wooden boxes (10×10×10 cm) as previously described [10]. Single brood frames contained more than 2000 bees that emerged along a two-week period. Then, we obtained about 20 cages per comb with bees presenting low genetic variations to be used in each experimental series. As a result, each treatment (or age class) was replicated three-four times. In order to guarantee that the differences observed in behavior were not due to rearing environmental differences (other than the experimental treatments), all the groups were assayed within a two-week period. Caged bees were fed with 1.8M sugar solution, water and pollen *ad libitum* and kept at 30°C, 55% relative humidity and darkness until 17 days of age, when they were captured for testing.

### Testing and Conditioning of the Proboscis Extension Response

The olfactory learning behavior was analyzed using the Proboscis extension response (PER) paradigm [19], [20]. To evaluate the PER, bees were harnessed after anesthesia at -4°C and kept in an incubator (30°C, 55% relative humidity and darkness) for 3 hours. Then, only those bees that showed the unconditioned response (UR) after applying 1.8M sucrose solution onto the antennae were tested.

To assay the PER, a device that delivered a continuous airflow (50 ml/s) was used for the application of the odorant. Four microliters of pure odorant impregnated on 30×3 mm filter paper inside a syringe was delivered through a secondary air-stream (6.25 ml/s) to the head of the bee. A fan extracted the released odors to avoid contamination.

In most of the experiments, long-term memories were quantified during five testing events (CS presented without the US with an inter-trial interval of 10 min). When animals are exposed to a CS alone that has been previously paired with an US, the first stimulus presentation is commonly considered as a measure of memory retention. The successive presentation of the unrewarded CS causes a decrease in the CR level from the first to the last testing event and it is considered as a measure of extinction [18]. Only in *Caged bees exposed to a single odor*, subjects were assayed through a differential PER conditioning (see Experimental Procedure for details).

Both the testing events and the conditioning trials lasted 40 s. Before odor presentation, bees rested for 15 s in the airflow for familiarization as well as for testing their response towards the mechanical stimulus. Then, the odor was presented for 6 s and the responses were measured.

During the conditioning, bees were subjected to a standard differential protocol in which two pure odors were presented [20]: a rewarded one (rewarded conditioned stimulus, CS+) with 1.8M sucrose solution (unconditioned stimulus, US), and another non-rewarded one (non-rewarded conditioned stimulus, CS-). Bees were conditioned by four-rewarded and four non-rewarded learning trials (inter-trial interval of 10 min) in a pseudo-random order (+--++--+).

### Experimental Procedure

#### Caged bees fed with a scented sugar solution as an early olfactory experience

**Figure 1:**
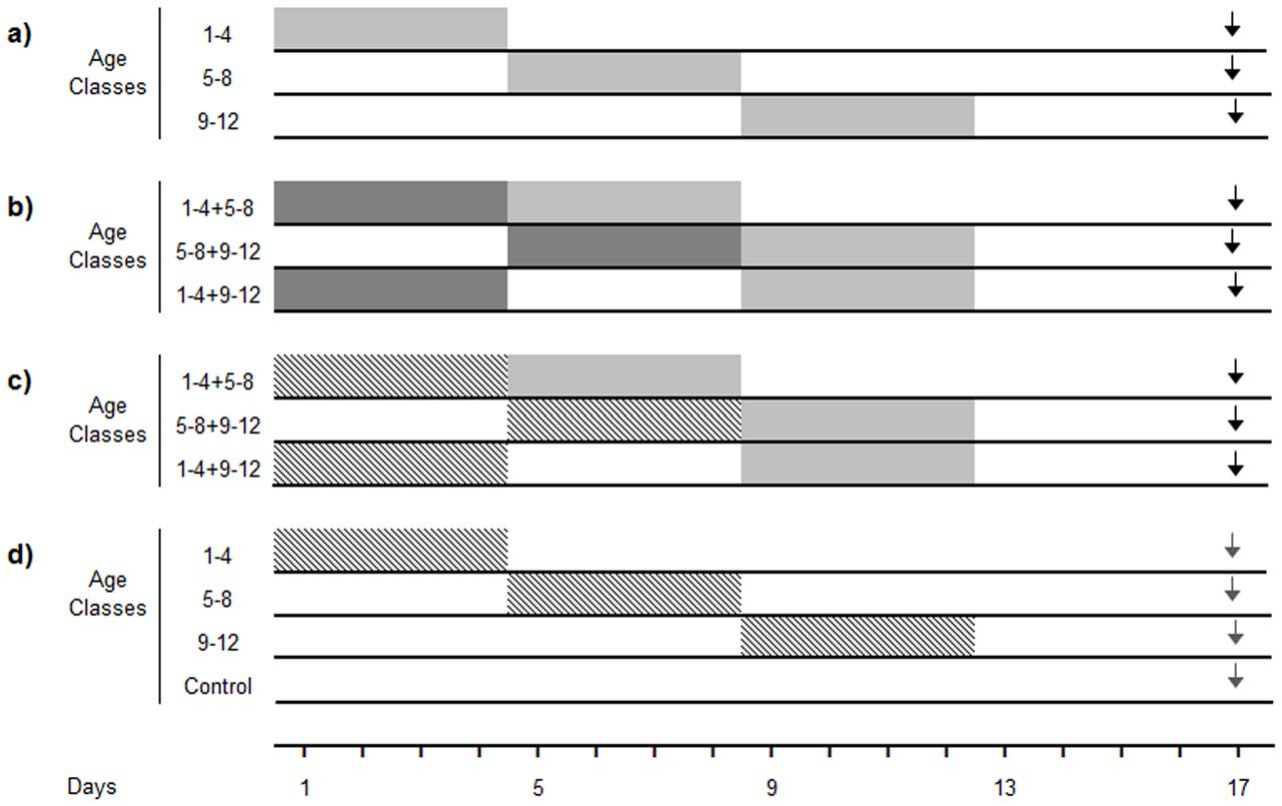
Schematic schedule of the experimental series along the adult lifespan of the honeybee. (**a**) Caged bees were offered a scented sugar solution for four consecutive days (gray boxes), and their olfactory memories at 17 days of age were evaluated in the PER paradigm (black arrow). (**b**) In addition to the scented solution received in a), bees were offered an alternative scented food for four consecutive days (1–4 or 5–8 days old, dark gray boxes). Then, bees were stimulated at: 1–4 + 5–8, 1–4 + 9–12 and 5–8 + 9–12 days of age. (**c**) An odor was exposed as volatile compound for four consecutive days (crossed boxes) before caged bees were offered the scented sugar solution (gray boxes). Odor exposure memory was not tested. (**d**) Caged bees were exposed to a pure odor for four consecutive days (crossed boxes) before conditioning (gray arrow). Notice that a non-odor exposed group was also included.

It has been shown that the offering of a scented sugar solution leads to long-term retention in honeybees and reduces the mortality as compared to the PER conditioning procedure [10]. The scented food was offered inside the cages when bees were 1–4, 5–8 or 9–12 days of age (Fig. 1a). These time windows were chosen arbitrarily [10]. However, they represent well-defined periods in which anatomical and functional changes in the honeybee interact with the development of the social activities in the hive. Immediately after emergence (1–4 days of age), the worker bee scarcely performs tasks [at least during the 24–48 hours as an adult [15]–[17]]; meanwhile its olfactory system is still in development. One week after emergence, worker bees start cleaning cells actively and taking care of the brood; at that time, the maturation of the olfactory system is finished. Middle-aged bees (i.e. 9–12-days-old-bees) handle and store food inside the hive, whereas their olfactory circuitry is already completed [21]–[23]. Within these periods, the scented food was always placed in the cages as the only source of sugar. The odors used were floral odors: Linalool (LIO) and Phenylacetaldehyde (PHE). The three treatments (1–4, 5–8 or 9–12 days) were repeated using either LIO or PHE. Odor solutions were obtained by mixing 50 µl of pure odorant per liter of 1.8M sucrose solution. To reduce volatiles inside the cages, the scented food was offered through plastic tubes (10 ml volume) with a small opening (1 mm diameter) in its end, which allowed bees to drink only one at a time.

We tested their long-term memories in five testing events (CS alone) in the PER assay when experimental bees were 17 days of age (Fig. 1a).

#### Caged bees fed with two different scented sugar solutions

In order to determine the effect of a prior olfactory appetitive experience on later associative learning, groups of bees were fed with scented food at 5–8 or 9–12 days of age and also with a second scented sugar solution at 1–4 and 1–4 or 5–8 days of age, respectively. As a consequence, the experimental groups 1–4 + 5–8, 5–8 + 9–12 and 1–4 + 9–12 days of age received scented food for 8 days (Fig. 1b). Memories established during the first 4 days were not tested. Whenever LIO was used as the rewarded odor, PHE was used as the non-rewarded one and vice versa. Again, multiple-PER tests to the CS were carried out in 17-day-old bees (Fig. 1b).

To avoid confounding generalization effects between groups that had been treated with either a single odor or multiple odors, we restricted comparisons within particular experiments (individuals that received only one scented food for a four-day period or that were exposed to the odor before the offering of scented food, etc; Fig. 1). Although we could not quantify the percentage of PER due to olfactory generalization between the odor used for the early experiences and for the conditioning, we assumed that this is similar in different age classes in each experimental series. Indeed, the odors used here have been reported to exhibit low generalization responses and no interactions with the age at which they were presented [10], [11].

#### Caged bees exposed to a volatile compound before offering the scented food

To separate the effects of a rewarded experience from the effect of the sensory stimulation we also exposed an odor as a passive volatile in the rearing environment of the bees. Following an experimental design similar to that in the previous experiment, we exposed caged bees to a volatile odor (non-tested exposed odor) as a preceding olfactory experience instead of the offering of scented food. Thus, bees conditioned at 5–8 and 9–12 days of age were also exposed to a volatile compound at 1–4 and 1–4 or 5–8 days of age, respectively (Fig. 1c). To carry out the odor exposure, caged bees were moved to another incubator (same conditions of temperature, relative humidity and darkness). There, cages were placed inside plastic boxes (80.5×48×35 cm), where 300 µl of pure odor was presented in filter papers (20 cm^2^ evaporation surface) located on the sides of the boxes. To reduce odor accumulation, an air extractor was connected to the incubator. After the odor exposure, cages were replaced by new ones and bees moved back to the first incubator to prevent odor contamination during non-exposure periods.

When LIO was used as the rewarded odor, PHE was used as non-tested exposed odor and vice versa. Seventeen-day-old bees were evaluated towards the odor solution but not to the exposed odor (putative olfactory memories established during the odor exposition were tested in the following experiment).

#### Caged bees exposed to a single odor in the environment as an early olfactory experience

As a control of the above experiment and to evaluate putative long-term effects of early volatile exposure over later learning, groups of 1–4, 5–8 and 9–12-day-old bees were exposed to a pure odor for four consecutive days inside the cages (Fig. 1d). A fourth group never exposed to the odor was used as control. For the olfactory exposure, we followed exactly the same procedure as that described in the previous section.

It has been observed that the exposure of honeybees to an odor in the environment leads to a slower rate of acquisition when the bee has to learn to associate the same stimulus with a reward [24], [25], a phenomenon known as latent inhibition (LI). Because this effect cannot be easily detected through a single PER test, we carried out a differential PER conditioning using the exposed odor either as a rewarded or unrewarded conditioned stimulus (CS+ and CS−, respectively). Then, when LIO was used as CS+, PHE was used as CS−, and vice versa.

### Statistical Analysis

The proboscis extension response (PER) was assayed using analyses of variance for repeated measurements (RM-ANOVA). Monte Carlo studies have shown that it is possible to use ANOVA on dichotomous data [26]. When we detected statistical differences in the principal factors, we carried out Fisher LSD *post-hoc* comparisons. To analyze PER values during differential conditioning, we used a discrimination index (DI) which is the difference between the sum of the positive responses towards the CS+ minus the sum of the positive responses towards the CS− for each honeybee for the whole conditioning (DI = ΣCS+ - ΣCS−). The DI was analyzed using one-way ANOVA.

## Results

### Caged Bees Fed with One Scented Sugar Solution as an Early Olfactory Experience

**Figure 2:**
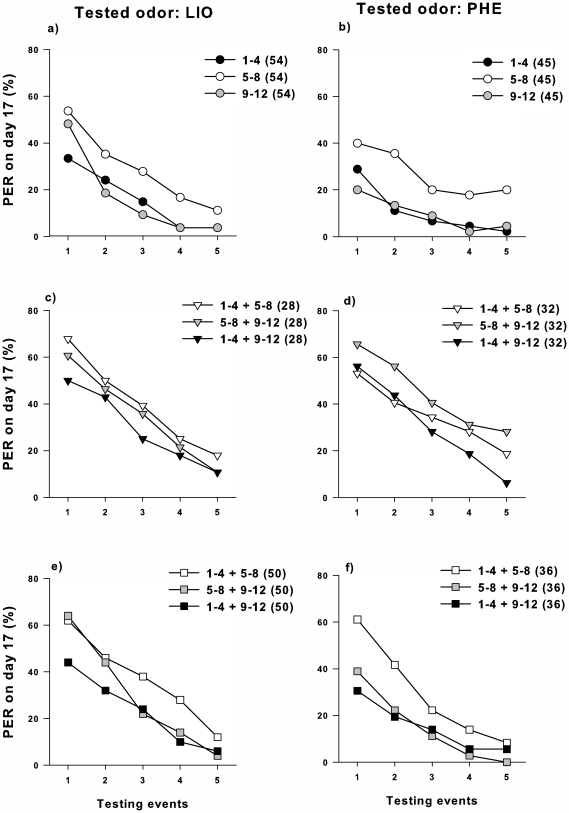
PER of early olfactory memories. Long-term memories were quantified during five testing events in the proboscis extension response (PER) assay in 17-day-old bees that were either offered scented sugar solutions or exposed to odors during an early period of their adult lifespan. LIO (left panel) or PHE (right panel) were used as the tested odors when LIO (**a**) or PHE (**b**) were offered alone in the sugar solution (*circles*); when PHE (**c**) or LIO (**d**) were the non-tested odors used to scent the alternative aromatized food (*triangles*); and when PHE (**e**) or LIO (**f**) were the non-tested odors used as volatiles exposed in the rearing environment (*squares*). Number of subjects is indicated in brackets at the legend.

Different early olfactory associative learning established by offering a scented sugar solution leaded to different extinction responses in 17-day-old bees. In each experimental series (LIO and PHE series), significant differences in PER were found for both factors: the testing events (Two-way RM-ANOVA LIO: F_4, 636_ = 57.66, p<0.001, PHE: F_4, 524_ = 17.31, p<0.001; Fig. 2a and 2b) and the age classes (Two-way RM-ANOVA: LIO: F_2, 159_ = 3.38, p = 0.036; PHE: F_2, 131_ = 5.08, p = 0.007; Fig. 2a and 2b). With the LSD *post-hoc* comparisons, we observed that responses of 5-8-day-old bees were statistically different from 1-4- and 9-12-day-old bees in both series (LSD comparison LIO: p_1-4 *vs* 5–8_ = 0.022, p_5–8 *vs* 9–12_ = 0.03; PHE: p_1–4 *vs* 5–8_ = 0.009, p_5–8 *vs* 9–12_ = 0.005; Fig. 2a and 2b). No differences were observable between bees fed with scented food at 1–4 and 9–12 days of age. The interactions between age classes and testing events were not significant (Two-way RM-ANOVA LIO: F_8, 636_ = 1.58, p = 0.127; PHE: F_8, 524_ = 0.99, p = 0.436; Fig. 2a and 2b). Then, our results showed that memory established in each experimental group was extinguished since a decrease in the percentage of the CR from the first to the fifth testing event was observed. However, we failed to detect statistical differences between extinction performances since the interaction factor age classes x events was not significant. Therefore, the memories established at different ages were caused at least by differences in CR after memory retrieval (retention), in which the highest retention levels were observed for the 5–8-day age class.

### Caged Bees Fed with Two Different Scented Sugar Solutions

When bees were offered two scented sugar solutions inside the cages, differences previously observed in *Caged bees fed with one scented sugar solution* between age classes were not detected by the analysis (Two-way RM-ANOVA, LIO: F_2, 81_ = 0.61, p = 0.547; PHE: F_2, 93_ = 1.19, p = 0.307; Fig. 2c and 2d). However, significant differences found among testing events (Two-way RM-ANOVA, LIO: F_4, 324_ = 32.97, p<0.001; PHE: F_4, 372_ = 24.77, p<0.001; Fig. 2c and 2d) revealed that extinction occurred. No statistical differences detected for the interactions between factors (Two-way RM-ANOVA, LIO: F_8, 324_ = 0.27, p = 0.974; PHE: F_8, 372_ = 0.52, p = 0.844) suggest that the dynamic of the extinction along the testing events between age classes are similar. These results strongly support the idea that early odor-rewarded stimulation prevents memorizing deficit observed in bees fed with a single scented food at 9–12 days of age.

### Caged Bees Exposed to a Volatile Compound before the Offering of Scented Food

As observed in bees fed with only one scented food solution, we also found differences in the CRs through the five tests between treatments when LIO was the volatile compound which preceded the offering of PHE-scented food (Two-way RM-ANOVA, PHE: F_2, 105_ = 3.42, p = 0.036; Fig. 2f). *Post-hoc* comparisons showed that responses obtained for 1–4 + 5–8 days of age were different from those of 5–8 + 9–12 and from 1–4 + 9–12-day-old bees as well (LSD comparison LIO: p _1–4+5–8 *vs* 5–8+9–12_ = 0.026, p _1–4+5–8 *vs* 1–4+9–12_ = 0.026, Fig. 2f). Once again, significant differences between testing events were found (Two-way RM-ANOVA, F_4, 420_ = 35.53, p<0.001; Fig. 2f), but no interaction was detected between the factors (Two-way RM-ANOVA, PHE: F_8, 420_ = 1.54, p = 0.142). The absence of interaction suggested that differences in the response performance seem to be caused by differences in memory retention established at different ages and not in differences in their extinction response.

On the other hand, groups that were exposed to PHE in the rearing environment and in turn fed with LIO-scented food did not differ in their performances (Two-way RM-ANOVA LIO: F_2, 147_ = 2.21, p = 0.114; Fig. 2e). Thus, the effect of the odor exposure on memorizing depended, at least weakly, on odor identity and the timing of the exposure. Again the two-way RM-ANOVA revealed significant differences between events (LIO: F_4, 588_ = 66.96, p<0,001; Fig. 2e). No interactions were detected in this experiment (Two-way RM-ANOVA, LIO: F_8, 588_ = 1.72, p = 0.091).

### Caged Bees Exposed to a Single Odor in the Environment as an Early Olfactory Experience

**Figure 3:**
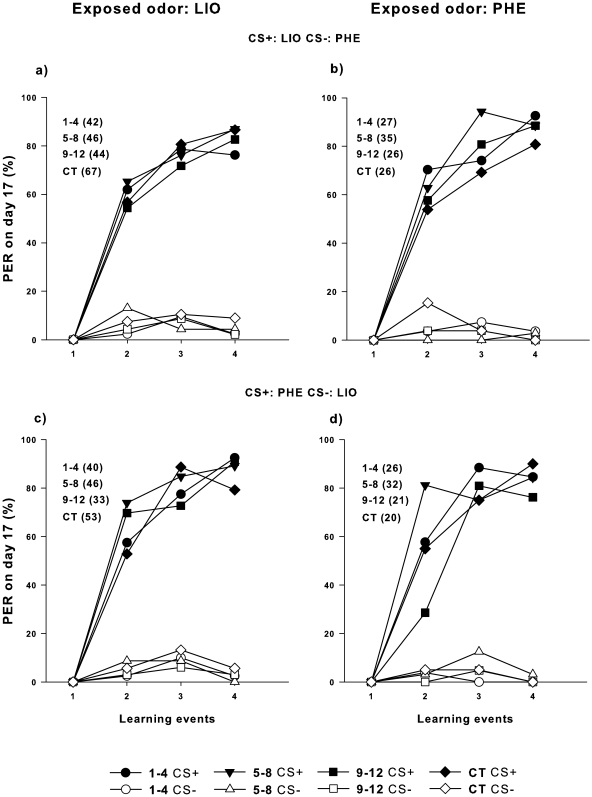
Differential conditioning after early odor exposure. The learning performance was quantified during four conditioning trials in the proboscis extension response (PER) assay in 17-day-old bees that were exposed to a single odor as volatile in the rearing environment during different age periods: 1–4 days of age (*circles*); 5–8 days of age (*squares*), 9–12 days of age (*triangles*) and controls (*rhombuses*); We used closed symbols for CS+ and open ones for CS−. LIO as the rewarded odor (CS+) and PHE as the non-rewarded odor (CS−) were used in the differential conditioning when LIO (**a**) or PHE (**b**) were early exposed. PHE as the rewarded odor (CS+) and LIO as the non-rewarded odor (CS−) were used in the differential PER conditioning when LIO (**c**) or PHE (**d**) were early exposed. Number of subjects is indicated in brackets at the legend.

We analyzed the long-lasting effect of odor exposure on subsequent learning. The similar discrimination indexes (DIs) found in bees exposed at different periods to LIO and PHE and even between exposed and control bees (non-exposed bees) revealed no long-lasting effect of the passive olfactory experience (One-way ANOVA: LIO_CS+LIO/CS-PHE_: F_3, 195_ = 0.079, p = 0.971, Fig. 3a; PHE_CS+LIO/CS-PHE_: F_3, 110_ = 1.892, p = 0.134, Fig. 3b; LIO_CS+PHE/CS-LIO_: F_3, 168_ = 0.976, p = 0.405; Fig. 3c PHE_CS+PHE/CS-LIO_: F_3, 95_ = 1.081, p = 0.360; Fig. 3d, see also Fig. S1 in Supplementary material).

## Discussion

Our results show for the first time that relatively brief olfactory stimulations at the early stages of the adult bee's lifespan can improve the memorizing of new learning events. Early associative learning either at the first four days of adulthood or at 5–8 days of age clearly modified the level of retention of long-term memories acquired thereafter (Fig. 2c and 2d) but might also influence extinction performance (not detected in our analysis). The 9-12-day-old conditioned bees that were previously stimulated with a scented sugar solution elicited higher retention when tested at 17 days of age as compared with those conditioned at the same age but without an additional olfactory experience. Moreover, the level of the initial conditioned response (CS-US memory retention) of the 9–12 days groups with or without precocious experience also suggested differences in retrieval according to the identity of the conditioned odor (e.g. the difference between Fig. 2a and 2c was 2–12%, while that between Fig. 2b and 2d was about 36–45%).

On the other hand, volatile pre-exposure seems to also improve retention of long-term memories acquired at middle ages (9–12 days), although its effect was weaker than the one found for the odor-rewarded experiences and cannot be extended to the both volatile compounds used (but only for PHE). In addition, it is fairly possible that such specific effect also depends on the interaction with the exposure timing. PER values appeared to be enhanced when PHE was exposed immediately before conditioning at 5–8 days of age but not before (Fig. 2e). In line with these thoughts, no long-term effect was detected during differential conditioning in 17-day-old bees pre-exposed to a single odor in the environment (Fig. 3). However, clear evidences about short-term and mid-term effects in young bees have been found both when individuals were exposed to volatiles in the PER setup [24] and inside the hive [11]. Then, our results support the idea that although exposed odors offered to early and middle-aged bees are able to promote a learning-reduced effect (LI), it would be restricted to the short/middle-term.

Both the rewarded and non-rewarded olfactory experiences might facilitate learning and, in turn, retention of the same type of information at a later time. However, the different outcomes for each type of odor experience suggest different mechanisms by which memories established at middle adult ages are better retained. It is well known that newly emerged bees are able to acquire and store odor-rewarded information in an associative manner [10], [13], a process that induces changes in the olfactory circuits of the antennal lobe, affecting both the processing and consolidation of odor information [27], [28]. This suggests that putative learning-induced changes in the brain of young adult bees might strengthen olfactory pathways that allow not only the retrieval of a specific piece of information acquired at very early ages (Fig. 2a and 2b) but also that acquired later in life. In this sense, a recent study has shown that early odor learning during young adulthood, between 5 to 8 days after emergence, affects neural activity in the honeybee antennal lobe on a long-term scale [29]. Conditioned odors evoke enhanced glomerular activity and modify spatiotemporal response patterns, which would underlie long-term olfactory memory for the early-experienced odor, but also for learning-induced modifications that persist in the brain, allowing bees to better retrieve the information they have acquired a few days before.

As no memories were detected when the bees of the different ages studied were exposed to volatile compounds (Fig. 3), the slightly enhanced response in 5–8 + 9–12-day-old bees may not account for learning-induced persistence changes in the brain. However, volatile exposure could still alter the normal development of the olfactory system, as has been shown for the exposure of queen mandibular pheromone, which at a young age can affect the workers' aversive learning abilities [30]. Alternatively, PHE exposure at 5–8 days of age might sensitize the bees in the short term, a process that would facilitate the acquisition and retention of new learning events. On the other hand, LIO rather than PHE used as conditioned stimulus at 9–12 days of age might be responsible for our results. Asymmetries in retention of memories between LIO and PHE have been reported in honeybees [11]–[12], [31] but also in moths [32] and bumblebees [33] and might be related to the biological meaning of these odorants.

Our results strongly suggest that the olfactory experiences acquired during the first week of life within an appetitive context (and probably also some pure odor acquired in the environment) are critical for the development of the long-term memory at least in the adult bees conditioned immediately after this stage. Since the functional properties of the olfactory system have been shown to be strongly influenced by changes in the environmental conditions from 3 days before to 4–8 days after emergence [21], early odor experiences might be important for the correct maturation of the olfactory pathways. Hence, it seems plausible that young worker bees need to be subjected to a constellation of chemosensory stimuli, like odors in the food or in the rearing environment, to achieve proper learning and retention abilities at older ages [21]–[22], [34].

Within the hive, bees are constantly exposed to diverse scents. As foragers bring different types of scented nectar back to the nest, young workers can learn food odors through mouth-to-mouth trophallaxis [35]–[37] while food is shared among hive mates. On the other hand, one-week-old bees performing tasks such as nursing or food processing handle food very often and thus have the opportunity to associatively learn different floral odors directly from the honey cells [38]. Learning processes along the in-hive period might prepare workers for later tasks, including those performed outside the hive.

It also seems that the odors experienced by one-week-old bees will be later prioritized when these bees become foragers (Fig. 2a and 2b). Indeed, these experiences might bias food preferences in the field [31]. In the complex scenery of olfactory stimuli that exists within the hive, the prevailing rewarded odors that change frequently as a result of switching blooms could form memories accessible to retrieval later in life. Then, changes in the floral availability would be better addressed and resumed when bees with different early-induced olfactory preferences coexist within the colony. This would enhance the chances of a bee hive to respond efficiently to a changing floral offer.

Several authors argue that a rearing environment plenty of stimuli must be the natural situation in animal development, whereas deprivation of stimuli is often an artificial situation leading to behavioral abnormalities [39], [40]. We observed that early olfactory rewarded experiences prevented deficits in memories established at middle-age when the failure of a proper rearing environment might delay the maturation of the olfactory system. Then, in the honeybee, stimulus deprivation represents a non-natural situation that unveils that the retention of olfactory memories is age-dependent.

In summary, odor-rewarded experiences occurring within the first week of the adult lifespan bias odor-mediated responses to the previously experienced odorants at older ages, and enhance memorizing new learning events acquired later in life. These findings are not strictly reward specific, since a similar trend, although weaker, was also elicited by the exposure to certain odors.

## Supporting Information

Figure S1 Discrimination Index after early odor exposure. Discrimination index (DI) was calculated for four conditioning trials in the proboscis extension response (PER) assay for each single bee. The indexes were quantified in 17-day-old bees that were exposed to a single odor as volatile in the rearing environment during different age periods (1–4; 5–8; 9–12 days of age and control bees). LIO as the rewarded odor (CS+) and PHE as the non-rewarded odor (CS) were used in the differential conditioning when LIO (a) or PHE (b) were early exposed. PHE as the rewarded odor (CS+) and LIO as the non-rewarded odor (CS-) were used in the differential PER conditioning when LIO (c) or PHE (d) were early exposed. Number of subjects is indicated at the bottom of each bar. No statistical differences were detected (see in text for details).
(1.93 MB EPS)
